# Timing of troponin T measurements in triage of pulmonary embolism patients

**DOI:** 10.3325/cmj.2013.54.561

**Published:** 2013-12

**Authors:** Nikola Bulj, Ines Potočnjak, Mirella Sharma, Hrvoje Pintarić, Vesna Degoricija

**Affiliations:** 1 University of Zagreb School of Medicine, Zagreb, Croatia; 2 Department of Medicine, “Sestre Milosrdnice” University Hospital Center, Zagreb, Croatia; 3 University of Zagreb School of Dental Medicine, Zagreb, Croatia

## Abstract

**Aim:**

To determine the appropriate timing of cardiac troponin T (cTnT) measurement for the early triage of pulmonary embolism (PE) patients.

**Methods:**

In this single-center prospective study, PE was confirmed in all patients using computed tomography. 104 consecutive patients were divided into three groups (high-risk, intermediate, and low-risk) based on their hemodynamic status and echocardiographic signs of right ventricular dysfunction. cTnT levels were measured on admission and then after 6, 24, 48, and 72 hours with threshold values greater than 0.1 ng/mL.

**Results:**

Intermediate-risk PE patients had higher cTnT levels than low-risk patients already in the first measurement (*P* = 0.037). Elevated cTnT levels significantly correlated with disease severity after 6 hours (intermediate vs low risk patients, *P* = 0.016, all three groups, *P* = 0.009).

**Conclusion:**

In hemodynamically stable patients, increased cTnT level on admission differentiated intermediate from low-risk patients and could be used as an important element for the appropriate triage of patients.

Pulmonary embolism (PE) still remains one of the most frequent causes of death among patients in emergency settings. Early identification of high-risk patients and optimal treatment administration is often difficult due to the clinical variability of the disease. Several risk-stratification tools have been recently proposed based on echocardiography, biomarkers (troponins and natriuretic peptides), and computed tomography (1). Right ventricular dysfunction (RVD) is a central hemodynamic event in PE patients, and represents an independent prognostic factor of adverse events. Multiple clinical studies have convincingly shown that RVD in hypotensive and normotensive PE patients directly affects early mortality (2). A meta-analysis by ten Wolde et al clearly indicates that RVD is associated with a two times higher risk of PE related mortality (3). According to the current European Society of Cardiology (ESC) guidelines, therapy should be tailored to the estimated risk of death due to acute pulmonary embolism (4). The guidelines divide PE patients into three groups: high-risk (PE related mortality risk >15%), intermediate (PE related mortality risk 3%-15%), and low-risk patients (PE related mortality risk <1%) based on the presence of right ventricular dysfunction (measured by echocardiography, computed tomography, and natriuretic peptides) or injury (troponins). Higher risk patients should be treated with thrombolytic therapy in an intensive care unit (ICU), while non-high-risk patients should be additionally stratified and treated either in an ICU (intermediate-risk) or clinical ward (low-risk patients).

Laboratory evaluations of acute right ventricular injury in PE patients have been extensively performed over the past few years, with a special emphasis on the prognostic value of serum troponins due to their wide use in the routine evaluation of patients with chest pain. Elevated levels of serum troponin are found in approximately 30%-50% of patients with pulmonary embolism (5, 6). Despite low diagnostic accuracy, the prognostic value of elevated troponin levels in PE patients is well documented, and according to one study, is associated with a 3.5 times higher risk of total mortality during 3 months follow-up (4,7). In PE patients, the increase in serum troponin is usually moderate and its retention in plasma is shorter (up to 3 days) than in patients with acute coronary syndromes (8).

In an emergency department setting, patients with established PE should be properly stratified and consequently admitted either to an ICU or medical ward according to the presence of RVD or right ventricular injury. However, the optimal timing for troponin measurement has not been established due to methodological differences in clinical trials where different times for troponin sampling have been used (4) It is not clear whether the initial measurement of cardiac troponin in emergency department is enough for early clinical triage, or if subsequent control measurements are required in order to distinguish patients who need ICU or clinical ward treatment and surveillance. Thus, the aim of the present study is to investigate the appropriate timing of cardiac troponin T (cTnT) measurement for the early triage and follow up of pulmonary embolism patients.

## Patients and methods

This single-center prospective longitudinal observational study of a cohort of PE patients was conducted from September 2009 to November 2011 in the medical intensive care unit (ICU) at the “Sestre Milosrdnice” University Hospital Center in Zagreb, Croatia. The study population included 109 consecutive patients. In all patients the diagnosis of PE was confirmed by contrast enhanced spiral computed tomography during the first 3 hours after first admission to the emergency department. After ICU admission, complete data on baseline clinical, hemodynamic, and laboratory parameters were obtained using a standardized questionnaire by an intensive care specialist. All patients gave their informed consent. “Sestre Milosrdnice” University Hospital Center committee on human research approved the study protocol as a non-interventional study. Patients were excluded from analysis if they met at least one of the following criteria: (i) denial of preliminary consent or withdrawal of previously given consent for participation in the study; (ii) severe renal insufficiency or failure, defined as an estimated glomerular filtration rate (eGFR) of <30 mL/min/1.73m^2^ on admission; and (iii) left ventricular systolic dysfunction (ejection fraction <40%) or severe mitral valve disease as a potential cause of pulmonary hypertension and right heart disease. The study protocol strongly recommended a transthoracic echocardiogram within 24 hours of PE diagnosis. Right ventricular dysfunction was defined by the presence of at least one of following criteria: dilatation of the right ventricle (an end-diastolic diameter >30 mm from the parasternal view or a right/left ventricle diameter ratio >1.0 from the subcostal or apical view), septal systolic flattening, right ventricular free wall hypokinesis, or a tricuspid regurgitation jet >2.7 m/s.

**Figure 1:**
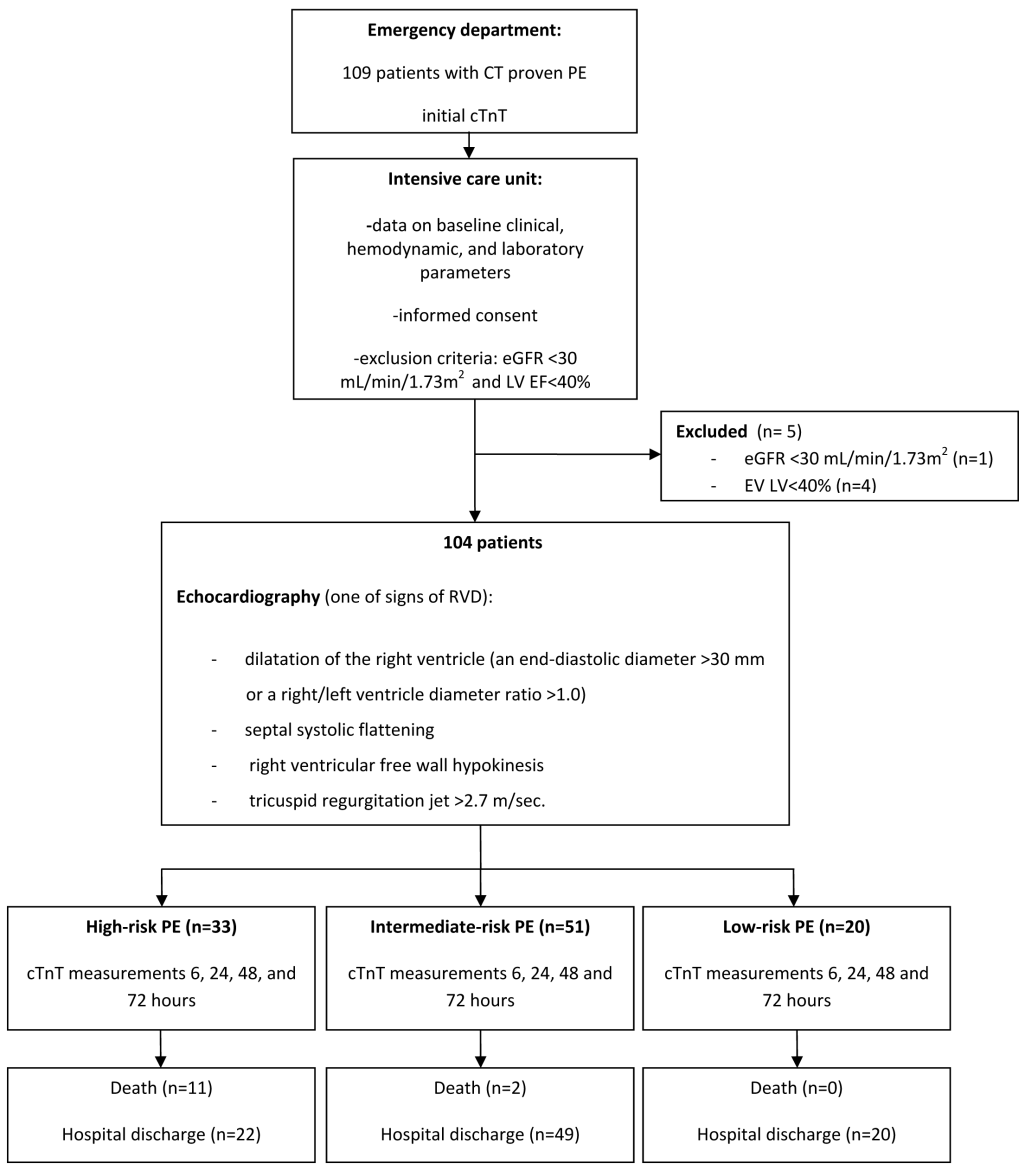
Study protocol. PE – pulmonary embolism; CT - computed tomography; cTnT – cardiac troponin T; eGFR – estimated glomerular filtration rate; LV EF – left ventricular ejection fraction; RVD – right ventricular dysfunction.

Patients were, according to their baseline short-term mortality risk, stratified into three severity groups: patients with high-risk (hemodynamically unstable patients defined as having a systolic blood pressure of <90 mm Hg, or a drop in systolic blood pressure of ≥40 mm Hg from baseline for a period >15 minutes); intermediate-risk (hemodynamically stable patients with a systolic blood pressure of >90 mm Hg and echocardiographic signs of RVD); and low-risk (hemodynamically stable patients with a systolic blood pressure of >90 mm Hg and without echocardiographic signs of RVD). Patients were followed up until hospital discharge or death (Figure 1).

Blood sampling for cTnT was done similarly to the protocol used for acute coronary syndromes as recommended by the European Society of Cardiology guidelines (9, 10). The study protocol recommended cTnT level measurement from blood samples obtained initially in emergency department and then after 6 hours in order to rule out initial “false negative” findings. Additional measurements, in accordance with the study protocol, were obtained after 24, 48, and 72 hours after admission in order to determine peak levels of the marker. cTnT was determined in serum by highly sensitive and specific electrochemiluminescent immunoassay (cTnT, Cat. No. 05092728) using the analyzer Elecsys 2010. Reagents, calibrators, and control samples, as well as the analyzer were all from the same manufacturer (F. Hoffmann-La Roche Ltd, Basel, Switzerland). The lower limit of detection for cTnT was 0.01 ng/mL. Based on the reference values of our laboratory, plasma concentrations of cTnT>0.1 ng/mL were considered to indicate myocardial injury and were used as the threshold value.

The eGFR was estimated using the Modification of Diet in Renal Disease (MDRD) study equation; renal insufficiency/chronic kidney disease was defined as GFR 30-60 mL/min/1.73 m^2^ body-surface area, and severe renal insufficiency or kidney failure as GFR<30 mL/min/1.73 m^2^ (11). Serum creatinine was measured by the Jaffe method (Modular P, Roche Diagnostic, Mannheim, Germany) (12) .

### Statistical analysis

To determine the sample size, an initial power analysis for χ^2^ test was performed with the following parameters: estimated effect size w = 0.4, α significance level 0.05, statistical power of 0.95, and 3 investigated groups (high risk, intermediate risk, and low risk PE). Sample size needed to meet these demands was 97. In total, 104 participants were included to account for possible drop outs or missing data.

Data were analyzed with STASTICA software, version 10.0 (StatSoft, Inc. Tulsa, OK, USA). Continuous variables normality of distribution was assessed with Kolmogov-Smirnov test. Continuous variables are presented as the mean ± standard deviation (SD) or median with interquartile range (IQR) according to data distribution. Box-and-Whisker plot represents medians with corresponding interquartile ranges, minimal and maximal values (excluding outliers). Data for variables assessed on nominal or ordinal scale were presented in contingency tables and analyzed by χ^2^ test (or Fisher exact test where appropriate – 2 by 2 table). The Mann-Whitney U test was used to compare differences in continuous variables between two groups (cTnT<or >0.1 ng/mL) and Kruskal-Wallis test was used for comparisons between three severity groups (high, intermediate, and low-risk PE). Differences were considered significant if the *P* value was <0.05.

## Results

A total of 109 consecutive patients were admitted to the “Sestre Milosrdnice” University Hospital Center with confirmed diagnosis of PE by computer tomography. Five patients (4.0%) were excluded from further analysis due to (i) renal failure with a GFR of <30 mL/min/1.73 m^2^ (n = 1), or (ii) left ventricular dysfunction with EF<40% (n = 4). None of the patients withdrew his/her consent. Thus, the final study population comprised 104 patients with acute PE. The patients had a mean age of 68.7 ± 13.4 (range, 25-89 years) and there was a female predominance (63.5%). There were 13 (12.5%) in-hospital deaths on the first day of hospitalization, and most of these were in the group with high-risk PE (11 patients). There were 33 (31.7%) patients in the high-risk, 51 (49.1%) in the intermediate-risk, and 20 (19.2%) in the low-risk group (Table 1). All patients were treated with low molecular weight heparin (enoxaparin 1 mg/kg subcutaneously every 12 hours), and no one received thrombolytics.

Most frequent clinical characteristics of the patients were dyspnea, chest pain, and tachycardia (Table 1). When compared with hemodynamically stable patients, syncope and shorter duration of symptoms (less than 12 hours) were more frequent in high-risk patients. Also eGFR<60 mL/min/1.73 m^2^ was observed more frequently in patients with high- and intermediate-risk PE than in low-risk PE patients.

Positive values of cTnT were detected in 29 of the 104 investigated patients (27.80%), with cTnT value already positive in 26 patients in the emergency department. Three of the initially positive cTnT patients in the high-risk group died and 3 initially cTnT negative patients reached cTnT>0.1 ng/mL 6 hours after admission. There were no new positives in the intermediate and low risk groups. Troponine positive patients were more frequently tachycardic (*P* = 0.034) and had right ventricular dysfunction (*P* = 0.029) (Table 2). Although the proportion of cTnT positive patients in high-risk group reached borderline significance (*P* = 0.059), this was observed in the first control measurement after 6 hours (*P* = 0.016). Control measurements of cTnT at 24, 48, and 72 hours after admission revealed no significant correlation with disease severity.

Fifteen (21.1%) out of 71 hemodynamically stable patients (intermediate and low risk group) had cTnT levels above 0.1 ng/mL. A significant proportion of cTnT positive patients was found in the intermediate-risk group in the first emergency department measurement (*P* = 0.037). The proportion remained significant in the first control measurement 6 hours after admission (*P* = 0.009). This was not observed in subsequent control measurements 24, 48, and 72 hours after admission.

**Figure 2:**
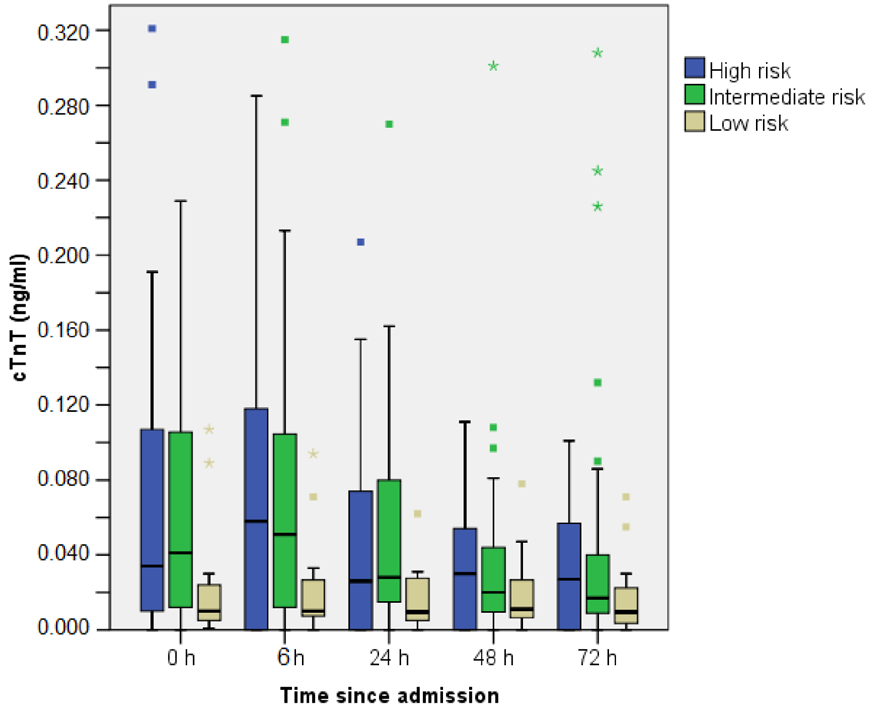
Cardiac troponin T kinetics in all investigated patients. Itis positive in 29 (27.8%) patients with peak levels observed at 6h.

On average, the first cTnT value was already positive on admission. cTnT peak occurred on average 6 hours after admission, and levels subsequently decreased (Figure 2).

## Discussion

The present study showed that when assessing PE patients in the emergency department two consecutive assessments of cTnT at an interval of 6 hours allowed for the identification of PE patients who required ICU admission and aggressive treatment. Pulmonary embolism is a medical emergency with a high mortality rate that in untreated patients reaches 30%. This is mainly due to repeated thromboembolism a few hours following the initial event (13, 14). Early detection and accurate diagnosis of high-risk patients is therefore of crucial importance for an appropriate treatment strategy and mortality reduction. Hemodynamic status at the time of admission is the most important prognostic factor of short-term mortality (15). High-risk pulmonary embolism or hemodynamically unstable pulmonary embolism is usually defined by the presence of circulatory shock and a mortality rate above 15%. On the other hand, a vast majority of patients are hemodynamically stable and can be considered to have non-high-risk pulmonary embolism. The mortality rate for this group ranges from 1%-15%. This large range reflects a wide spectrum of severity, ranging from trivial pulmonary embolism to pulmonary embolism with compensated shock that could deteriorate at any moment. In order to identify hemodynamically stable patients with increased risk of PE related complication, several risk-stratification tools were proposed based on clinical scores, imaging tools (CT and echocardiography), and biomarkers (troponins and natriuretic peptides) (16, 17).

Elevation of cTnTs during acute PE occurs in 13%-50% of patients (18, 19). cTnT is a highly sensitive and specific marker of myocardial necrosis and is the current gold standard biomarker for diagnosing myocardial infarction (20). During the acute phase of PE, the sudden rise in pulmonary vascular resistance secondary to pulmonary thrombi can lead to acute pressure overload of the right ventricle. Coupled with hypoxia and right coronary hypoperfusion due to systemic hypotension, there ensues myocardial ischemia/necrosis. In the last decade, several smaller studies have shown that elevation of cTnTs during acute PE is associated with increased in-hospital and short-term mortality (21, 22). A meta-analysis of 20 clinical trials showed that elevated levels of troponin in patients with PE were associated with an increased risk of early mortality, which applies both to hemodynamically unstable and stable patients (23). Thrombolytic treatment, usually performed in cases of severe PE with hemodynamic instability with right ventricular dysfunction and/or injury, may offer a life-saving therapy. Moreover, in a multicentric randomized trial of thrombolytics in normotensive PE patients (PEITHO trial), positive troponin was one of the main criteria for patient selection (24). On the other hand, in low-risk patients thrombolytic therapy is contraindicated, and anticoagulant therapy can be administered in hospital wards only. Furthermore, according to recent studies, some low-risk PE patients could be treated as outpatients (25, 26). Since the availability of a cardiac ultrasound on a round-the-clock basis can be a significant problem in some hospitals, and for some patients an echocardiographic exam can be difficult to perform in emergency settings, cTnTs could play an important role in the early clinical triage of PE patients.

Despite having good prognostic value, the appropriate timing for troponin measurement in PE patients is still unclear. The only study dealing with this issue included 200 hemodynamically stable PE patients and measured cardiac troponin I (cTnI) at admission and then every 8 hours following admission over a three-day period (27). They found that 15% of patients at the time of admission were misclassified as cTnI negative. The highest cTnI level was consistently reached 8 hours after initial presentation. The authors concluded that the first measurement of cTnI was a “false negative” because of a delay in the release of troponins after right heart injury. Two consecutive assessments of cTnI at an 8-hour interval allowed for the correct risk stratification of patients suffering from PE and patients with increased cTnI on admission, and a further blood sample for cTnI assessment was deemed unnecessary (27). Our study analyzed cTnT and suggested that initial “false negative” cTnT levels occurred only in hemodynamically unstable patients with shorter symptom durations. In unstable patients who survived the initial event, cTnT reached positive levels after 6 hours following admission. On the other hand, in hemodynamically stable patients with intermediate-risk PE, cTnT levels were found positive at the time of admission and remained positive for the next 6 hours. Furthermore, our data showed that, unlike patients with acute coronary syndromes, the most informative levels of cTnT were reached 6 hours after admission, with decreased values in subsequent measurements, and without the need for further blood sampling for cTnT measurement.

Although the final number of included participants complied with the sample calculation in power analysis, we see a potential drawback in different sizes of three analyzed groups. In our opinion, any future investigations should incorporate a larger and more balanced sample of patients, ideally using a multi-center design. This study was not designed to evaluate differences in mortality, still it might have some important clinical implications regarding the early triage of PE patients.

In conclusion, when assessing PE patients in the emergency department, two consecutive assessments of cTnT at a 6-hour interval should allow for the identification of PE patients who require aggressive ICU treatment and surveillance. In hemodynamically stable patients, an increased cTnT level on admission distinguishes intermediate from low-risk patients and could be used for the appropriate triage of patients.

## 

**Table 1:** Baseline characteristics of 104 patients with acute pulmonary embolism (PE)

	Group			P*	*P^†^*
	high risk PE (N = 33)	intermediate risk PE (N = 51)	low risk PE (N = 20)		
Female/male sex: n (%)	22 (66.7)	34 (66.7)	10 (50.0)	0.193	0.380
	11 (33.3)	17 (33.3)	10 (50.0)		
Reported symptom onset <12h: n (%)	17 (51.5)	13 (25.5)	3 (15.0)	0.341	0.009
Reported symptom onset >12h: n (%)	16 (48.5)	38 (74.5)	17 (75.0)		
Symptoms: n (%)					
Dyspnea	32 (97.0)	48 (94.1)	15 (75.0)	0.022	0.014
Syncope	19 (57.6)	2 (3.9)	0 (0.0)	0.396	<0.001
Chest pain	22 (66.7)	30 (58.8)	6 (30.0)	0.029	0.028
Cough	18 (54.5)	22 (43.1)	8 (40.0)	0.81	0.49
Tachycardia	22 (66.7)	32 (62.7)	4 (20.0)	0.001	0.002
Malignant tumor	8 (24.2)	18 (35.3)	6 (30.0)	0.671	0.561
Previous history of deep vein thrombosis or PE	5 (15.2)	6 (11.8)	4 (20.0)	0.37	0.667
Immobilization within 14 d	14 (42.4)	18 (35.3)	3 (15.0)	0.092	0.116
Positive family history	1 (3.0)	1 (2.0)	1 (5.0)	0.486	0.788
Adiposity	8 (24.2)	17 (33.3)	6 (30.0)	0.787	0.673
Death: n (%)	11 (30.0)	2 (3.9)	0 (0.0)	0.369	<0.001
Cardiac troponin *t* > 0.1 ng/mL: 0h, n (%)	11 (33.3)	14 (27.5)	1 (5.0)	0.037	0.059
Cardiac troponin *t* > 0.1 ng/mL: 6h, n (%)	11 (33.3)	14 (27.5)	0 (0.0)	0.009	0.016
Cardiac troponin *t* > 0.1 ng/mL: 24h, n (%)	6 (18.2)	8 (15.7)	0 (0.0)	0.6	0.138
Cardiac troponin *t* > 0.1 ng/mL: 48h, n (%)	5 (15.2)	4 (7.8)	0 (0.0)	0.197	0.157
Cardiac troponin *t* > 0.1 ng/mL: 72h, n (%)	2 (6.1)	4 (7.8)	0 (0.0)	0.197	0.442
Age (years): median (IQR)	74.0 (64.0-79.0)	71.0 (62.0-78.0)	70.0 (56.0-75.5)	0.561§	0.459‡
Body mass index (kg/m^2^): median (IQR)	27.0 (25.0-30.0)	27.0 (24.0-31.0)	27.5 (25.0-30.0)	0.898§	0.930‡
Creatinine: median (IQR)	95.0 (84.0-106.0)	105.0 (95.0-125.0)	97.0 (72.5-104.0)	0.015§	0.005‡
Estimated glomerular filtration rate: median (IQR)	57.0 (49.0-71.0)	52.0 (42.0-64.0)	69.5 (53.5-77.5)	0.007§	0.012‡

*Differences between low and intermediate risk for PE.

†Differences between all three risk groups for PE.

‡Kruskal-Wallis test; IQR – interquartile range.

§Mann-Whitney U test; IQR – interquartile range.

**Table 2:** Positive and negative findings of cardiac troponin T (cTnT) in 104 patients with acute pulmonary embolism (PE)

	cTnT>0.1 ng/mL		P*
	negative N = 75	positive N = 29	
High risk PE: n (%)	20 (26.7)	13 (44.8)	0.024
Intermediate risk PE: n (%)	36 (48.0)	15 (51.7)	
Low risk PE: n (%)	19 (25.3)	1 (3.4)	
Female: n (%)	45 (60.0)	21 (72.4)	0.238
Male: n (%)	30 (40.0)	8 (27.6)	
Reported symptom onset >12 h: n (%)	52 (69.3)	19 (65.5)	0.708
Reported symptom onset <12 h: n (%)	23 (30.7)	10 (34.3)	
Symptoms: n (%)			
Dyspnoea	67 (89.3)	28 (96.6)	0.240
Syncope	13 (17.3)	8 (27.6)	0.243
Chest pain	38 (50.7)	20 (69.0)	0.092
Cough	38 (50.7)	10 (34.5)	0.138
Tachycardia	37 (49.3)	21 (72.4)	0.034
Malignant tumor	26 (34.7)	6 (20.7)	0.166
Previous history of deep vein thrombosis or PE	12 (16.0)	3 (10.3)	0.462
Immobilization within 14 d	27 (36.0)	8 (27.6)	0.415
Family history	2 (2.7)	1 (3.4)	0.831
Adiposity	23 (30.7)	8 (27.6)	0.758
RVD (Echo positive): n (%)	58 (85.3)	29 (100.0)	0.029
Death: n (%)	10 (13.3)	3 (10.3)	0.679
Age (years): median (IQR)*	75.0 (64.0-78.0)	67.0 (60.5-80.5)	0.446
Body mass index (kg/m^2^): median (IQR)*	27.0 (24.0-30.0)	27.0 (24.5-30.0)	0.791
Creatinine: median (IQR)*	99.0 (88.0-112.0)	99.0 (90.5-116.5)	0.960
Estimated glomerular filtration rate: median (IQR)	59.0 (49.0-71.0)	53.0 (44.5-69.5)	0.374

*Mann-Whitney U test; IQR – interquartile range; RVD – right ventricular dysfunction.
